# Global practice guidelines in rheumatology: a cross-sectional altmetric and citation analysis

**DOI:** 10.1007/s00296-025-05899-z

**Published:** 2025-06-05

**Authors:** Olena Zimba, Mariusz Korkosz, Fatima Alnaimat, George E. Fragoulis, Marlen Yessirkepov, Chokan Baimukhamedov, Burhan Fatih Kocyigit

**Affiliations:** 1 Department of Rheumatology, Immunology and Internal Medicine, University Hospital in Krakow, Krakow, Poland; 2 National Institute of Geriatrics, Rheumatology and Rehabilitation, Warsaw, Poland; 3 Department of Internal Medicine N2, Danylo Halytsky Lviv National Medical University, Lviv, Ukraine; 4 Department of Rheumatology and Immunology, Jagiellonian University Medical College, Jakubowskiego 2 Str., 30-688 Kraków, Poland; 5 Department of Internal Medicine, Division of Rheumatology, School of Medicine, University of Jordan, Amman, Jordan; 6 First Department of Propaedeutic and Internal Medicine, Joint Academic Rheumatology Program, National and Kapodistrian University of Athens, Athens, Greece; 7 Center for Life and Health Sciences, National Academy of Sciences Under the President of the Republic of Kazakhstan, Almaty, Kazakhstan; 8 South Kazakhstan Medical Academy, Shymkent, Kazakhstan; 9 Shymkent Medical Centre of Joint Diseases, Shymkent, Kazakhstan; 10 Department of Physical Medicine and Rehabilitation, University of Health Sciences, Adana City Research and Training Hospital, Kışla District, Dr. Mithat Özsan Boulevard, 4522. Street No:28, Yüreğir, Adana, Türkiye

**Keywords:** Rheumatology, Practice guidelines as topic, Social media, Bibliometrics, Altmetrics

## Abstract

Clinical practice guidelines are instrumental for managing rheumatic diseases, which are often chronic, multifaceted, and require evidence-based diagnostic and therapeutic approaches. This study assessed the societal and academic implications of global rheumatology practice guidelines. A cross-sectional altmetric and citation analysis was conducted to assess the implications of rheumatology practice guidelines. Practice guidelines published in *Annals of the Rheumatic Diseases* and *Arthritis & Rheumatology* (2000–2024) were retrieved through PubMed searches. A total of 127 guidelines were included in this study. On April 9, 2025, the Altmetric Attention Score (AAS), Mendeley bookmarking data, and citation metrics from the Scopus and Dimensions databases were recorded for each document. A significant rise in the volume of published guidelines over time was detected (p < 0.001 for the trend). Guidelines in *Annals of the Rheumatic Diseases* attracted more Scopus citations (median: 320) compared to *Arthritis & Rheumatology* (median: 145.5; p = 0.046); AAS values did not show a significant difference between the sources (p = 0.168). The analysis demonstrated statistically significant positive correlations between Scopus citation counts and several altmetric indices, including AAS, news outlets, and Facebook mentions, and Mendeley bookmarking counts (all p < 0.001). No correlations recorded for X (Twitter) mentions. This study reveals the implications of global rheumatology practice guidelines in view of their traditional and alternative metrics. To maximize the societal implications, renewed social media strategies are warranted to expand online visibility and academic outreach of global rheumatology practice guidelines.

## Introduction

Social media (SoMe) platforms, as described by the Medical Subject Headings (MeSH) are defined as “platforms that provide the ability and tools to create and publish information accessed via the Internet and are characterized by user-generated content, a high degree of interaction between creator and viewer, and easy integration with other sites” have emerged as powerful instruments for medical interactions, research spread, and public involvement [1].

In rheumatology, these platforms are increasingly utilized by physicians, patients, organizations, and journals to disseminate knowledge, advocate guidelines, and enhance networking [2, 3]. Findings from a global survey by the Emerging EULAR Network (EMEUNET), composed of young rheumatologists, reflect this growing trend. Of 233 respondents from 47 countries, 83% reported using at least one SoMe platform, and 71% for professional purposes. Half of the participants used SoMe for clinical practice, and nearly 50% for research [4].

Clinical practice guidelines are essential tools in modern rheumatology, where conditions are often complex and chronic and require evidence-based approaches. These guidelines aim to consolidate the most reliable information into clear, practical recommendations that assist health professionals in making informed diagnostic and treatment decisions [5, 6]. Practice guidelines not only support clinical care but also influence policies and quality improvement programs [7, 8].

Global professional associations in rheumatology, namely the American College of Rheumatology (ACR) and the European Alliance of Associations for Rheumatology (EULAR), have played fundamental roles in formulating these guidelines. These documents are drafted through methodologically rigorous and well-organized processes, encompassing systematic literature reviews, expert consensus meetings, and evaluation of recommendations based on their strength and level of evidence [9].

The growing focus on evidence-based medicine and standardized care underscores the importance of practice guidelines in view of their effectiveness, enforcement, and implications [10]. Although the guidelines are expected to change clinical practice, limited attention is given to assessing their societal and citation-wise implications, which may also reflect, to some extent, their real-world implementation and integration into clinical workflows. Citation analysis primarily quantifies academic influence by determining the frequency with which a document is referenced in scholarly literature. Altmetric analysis assesses broader societal engagement by monitoring online attention across various platforms, including news outlets, social media, and reference managers. Exploring these metrics offers insights into their global outreach and use [11, 12].

This study comprehensively explored the implications of rheumatology practice guidelines published in the official journals of EULAR and ACR—*Annals of the Rheumatic Diseases* (*Ann Rheum Dis*) and *Arthritis & Rheumatology* (*Arthritis Rheumatol*)—focusing on their alternative metrics (altmetics) and citations. The aim was to systematically evaluate the structure and dynamics of SoMe reflections, measured by Altmetric Attention Score (AAS), and the imlications of global rheumatology practice guidelines citation-wise. An in-depth analysis of these guidelines was performed to reveal the trends of sharing, discussing, and referring within public and academic platforms.

## Methods

### Study design and data source

Cross-sectional altmetric and bibliometric analysis of global rheumatology practice guidelines was conducted as part of this study. All relevant guidelines were retrieved from PubMed by applying the publication type filter “practice guideline”[pt] along with specific journal filters:

- “Ann Rheum Dis”[jour] AND “practice guideline”[pt]
- “Arthritis Rheumatol”[jour] AND “practice guideline”[pt]

A total of 96 and 34 results were collected, respectively. Following a duplication assessment, all identified records were confirmed as unique and subsequently incorporated into the analysis. Following the PubMed inquiry, essential bibliographic details, including the title, Digital Object Identifier (DOI), year of publication, and source, were meticulously retrieved and recorded in a structured Excel spreadsheet for organizational and analytical purposes.

### Inclusion and exclusion criteria

Only documents specifically identified as practice guidelines and published in the *Ann Rheum Dis* and *Arthritis Rheumatol* were included. Three guidelines were excluded due to unavailability of recorded AAS data, indicated by the phrase, “*Altmetric hasn’t picked up any sharing activity around this article yet*.” Consequently, the final analysis included only guidelines with quantifiable altmetric data.

### Altmetric data

AAS is a quantitative measure of the attention an academic paper receives across various online platforms. Unlike traditional citation metrics, AAS captures real-time and publicly available engagement, including how often and in what context an article is mentioned online. AAS values were obtained using AltmetricIt tool. AAS is produced through an automated system that assigns different weights to various sources of online attention. Each source category is assigned a specific weight in accordance with Altmetric’s scoring algorithm. The score does not indicate quality or imply scientific importance but rather represents online presence, outreach, and engagement [13, 14].

Altmetric statistics were acquired utilizing the AltmetricIt program, which provides aggregated and source-specific metrics for each guideline. On April 9, 2025, the following data points were recorded for each published practice guideline:

- AAS
- Number of news outlets mentions
- Number of X (formerly Twitter) user mentions
- Number of Facebook mentions
- Mendeley bookmark count (signifies how many readers recorded the document in their personal Mendeley library).
- Dimensions citation count (the number of citations recorded in the Dimensions platform).

### Academic citation data

Citation metrics were gathered from the Scopus database to assess the impact of each guideline. A manual search using DOIs was conducted in Scopus. Scopus citations were recorded on April 9, 2025, aligning with the altmetric data collection date. This synchronization enabled direct correlation analysis between AAS and Scopus citation counts.

The five leading documents were identified based on their AAS and Scopus citation metrics to clarify the guidelines’ aggregated impact. This ranking approach focused on the most influential documents in view of their online (societal) implications and bibliometric impact.

### Statistical analysis

All statistical analyses were conducted using the Statistical Package for the Social Sciences (SPSS), version 23.0 (IBM Corp., Armonk, NY, USA). The Shapiro–Wilk test was utilized to assess the normality of data distribution. Given that the majority of variables did not meet the assumptions of normality, non-parametric statistical tests were employed. Data are presented as number (n), percentage (%), and median (minimum–maximum). The Mann–Whitney U test was employed to compare two independent groups. Continuous variables’ correlations were assessed using Spearman’s rank correlation coefficient (rho). Furthermore, a linear regression analysis was conducted to explore the trend in the annual count of practice guidelines over time. P < 0.05 was considered significant.

## Results

The PubMed search retrieved 130 guidelines. Three of these were excluded from the analysis due to unavailability of altmetric data. Therefore, 127 guidelines were included in the final analysis.

### Temporal distribution of guidelines

**Fig. 1:**
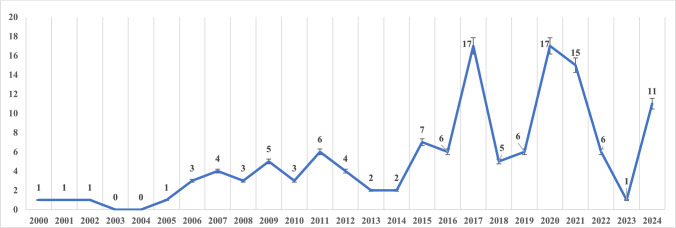
Distribution of clinical practice guideline numbers over the years

The earliest guideline was published in 2000. The annual number of guidelines increased, with notable peaks in 2017 and 2020, each including 17 documents. The linear regression analysis revealed a statistically significant upward trend (p < 0.001), indicating an average annual increase of 0.43. Figure 1 depicts the distribution of guidelines by publication year.

### Journal data

The *Ann Rheum Dis* published 93 (73.2%) of the analyzed documents, while the remaining 34 (26.8%) were published in *Arthritis Rheumatol*. A comparative analysis assessed practice guidelines’ citation and altmetric performance from the two rheumatology journals. Regarding Scopus citation counts, a statistically significant difference was detected between the two journals (p = 0.046):

- For *Ann Rheum Dis*, the median citation count was 320 (3–2073);
- For *Arthritis Rheumatol*, the median citation count was 145.5 (11–2590).

AAS values did not demonstrate a statistically significant difference between the journals (p = 0.168):

- For *Ann Rheum Dis*, the median AAS was 49 (3–514);
- For *Arthritis Rheumatol*, the median citation count was 74 (3–480).

### Influence of publication period on citations and altmetric data

A statistically significant difference was identified in the Scopus citation counts between practice guidelines published in the last decade and those published earlier (p = 0.002). In the past ten years, 91 guidelines were published, garnering a median citation count of 202 (3–2590). The earlier guidelines (n = 36) presented a median citation count of 529 (27–1751). Furthermore, a significant difference in the median AAS was detected between the two sets of guidelines (p < 0.001). The guidelines published in the last decade had a median AAS of 76 (3–480), whereas the older guidelines had a median AAS of 29.5 (3–514).

### Top-ranked guidelines according to the citation and altmetric data

Tables 1 and 2 present the five top guidelines, ordered according to Scopus citations and AAS. These documents are the most impactful and widely disseminated guidelines in the field of rheumatology.

**Table 1:** Top five practice guidelines in terms of Scopus citations

Title	Journal	Year	Citations count
2015 American College of Rheumatology Guideline for the Treatment of Rheumatoid Arthritis	*Arthritis Rheumatol*	2016	2590
EULAR recommendations for the management of rheumatoid arthritis with synthetic and biological disease-modifying antirheumatic drugs: 2019 update	*Ann Rheum Dis*	2020	2073
EULAR recommendations for the management of rheumatoid arthritis with synthetic and biological disease-modifying antirheumatic drugs: 2013 update	*Ann Rheum Dis*	2014	1751
Treating rheumatoid arthritis to target: recommendations of an international task force	*Ann Rheum Dis*	2010	1724
2019 update of the EULAR recommendations for the management of systemic lupus erythematosus	*Ann Rheum Dis*	2019	1501

**Table 2:** Top five practice guidelines in terms of altmetric attention score

Title	Journal	Year	AAS
EULAR recommendations for the management of rheumatoid arthritis with synthetic and biological disease-modifying antirheumatic drugs: 2013 update	*Ann Rheum Dis*	2014	514
2019 American College of Rheumatology/Arthritis Foundation Guideline for the Management of Osteoarthritis of the Hand, Hip, and Knee	*Arthritis Rheumatol*	2020	480
EULAR recommendations for the management of rheumatoid arthritis with synthetic and biological disease-modifying antirheumatic drugs: 2019 update	*Ann Rheum Dis*	2020	444
2015 American College of Rheumatology Guideline for the Treatment of Rheumatoid Arthritis	*Arthritis Rheumatol*	2016	359
2018 EULAR recommendations for physical activity in people with inflammatory arthritis and osteoarthritis	*Ann Rheum Dis*	2018	331

*AAS* altmetric attension score

The *2015 American College of Rheumatology Guideline for the Treatment of Rheumatoid Arthritis* is the most cited guideline, which is cited 2590 times (Table 1). Several EULAR guidelines on rheumatoid arthritis and systemic lupus erythematosus attracted 1501 to 2073 Scopus citations.

The *EULAR recommendations for the management of rheumatoid arthritis with synthetic and biological disease-modifying antirheumatic drugs: 2013 update* is the top-ranked guideline based on AAS, with a score of 514. This was closely followed by the *2019 American College of Rheumatology/Arthritis Foundation Guideline for the Management of Osteoarthritis of the Hand, Hip, and Knee* and *EULAR recommendations for the management of rheumatoid arthritis with synthetic and biological disease-modifying antirheumatic drugs: 2019 update*, with AAS values of 480 and 444, respectively.

Among the examined documents, three guidelines are topped by Scopus citations and AAS. These were the *2015 American College of Rheumatology Guideline for the Treatment of Rheumatoid Arthritis*, the *EULAR recommendations for the management of rheumatoid arthritis with synthetic and biological disease-modifying antirheumatic drugs: 2019 update*, and the *EULAR recommendations for the management of rheumatoid arthritis with synthetic and biological disease-modifying antirheumatic drugs: 2013 update.* Their simultaneous presence in citation and altmetric data underscores their combined influence, citation strength, and extensive dissemination and engagement on online platforms.

### Correlation analysis

Correlation analyses were conducted to investigate the links among Scopus citation count, Mendeley bookmarking count, and several altmetric indicators (Tables 3 and 4). Significant correlations were revealed between Scopus citation count and key indicators:

- AAS (rho = 0.424, p < 0.001);
- News outlets mentions (rho = 0.311, p < 0.001);
- Facebook mentions (rho = 0.474, p < 0.001);
- Mendeley bookmarking count (rho = 0.891, p < 0.001);
- Dimensions citation count (r = 0.998, p < 0.001).

**Table 3:** Correlations between Scopus citation count and altmetric data—Mendeley reader count—Dimensions citation count

Variables	AAS	News outlets mentions	X users mentions	Facebook mentions	Mendeley reader count	Dimensions citation count
Scopus citation count	0.424^*^	0.311^*^	0.099^**^	0.474^*^	0.891^*^	0.998^*^

*AAS* altmetric attension score; *p < 0.001, **p > 0.05

**Table 4:** Correlations between Mendeley reader count and citation—altmetric data

Variables	AAS	Scopus citation count	News outlets mentions	X users mentions	Facebook mentions	Dimensions citation count
Mendeley reader count	0.579^*^	0.891^*^	0.444^*^	0.318^*^	0.591^*^	0.894^*^

*AAS* altmetric attension score; *p < 0.001

No significant correlation was recorded between Scopus citations and X (Twitter) mentions (r = 0.099, p > 0.05).

Additionally, Mendeley bookmarking count was significantly correlated with the following parameters:

- AAS (rho = 0.579, p < 0.001);
- Scopus citation count (rho = 0.891, p < 0.001);
- News outlets mentions (rho = 0.444, p < 0.001);
- X (Twitter) posts (rho = 0.318, p < 0.001);
- Facebook mentions (rho = 0.591, p < 0.001);
- Dimensions citation count (rho = 0.894, p < 0.001).

## Discussion

### Key results

This study comprehensively examined rheumatology practice guidelines published in the *Ann Rheum Dis* and *Arthritis Rheumatol*, emphasizing their impact and online prominence through citation metrics and altmetric data. A total of 127 guidelines published in 2000–2024 were included in the analysis. The findings indicate a significant rise in the frequency of guideline publications and underscored significant differences in academic and altmetric performance of journals and publication periods. Moreover, citation metrics exhibited significant correlations with altmetric indicators and Mendeley bookmarking counts.

### Temporal trend

The linear regression analysis revealed a statistically significant annual increase in the number of guidelines, with an increment of 0.43 documents per year and noteworthy peaks in 2017 and 2020. This data aligns with the overall rise in rheumatology publications [15]. Additionally, rheumatic diseases are receiving increased attention because of their chronic, multisystemic, and often debilitating traits, requiring well-coordinated, evidence-based management strategies [16]. The increase in guideline numbers reflects a growing trend among health professionals to adhere to established evidence-based practice protocols for patient management, particularly in specialties known for their diagnostic and therapeutic complexity. As rheumatology rapidly evolves to include innovative medicines and personalized healthcare strategies, practice guidelines serve as both decision-making tools and a way to standardize practices across different healthcare systems [17].

### Journal-based comparisons

A significant difference in Scopus citation counts was found between the two journals, with *Ann Rheum Dis* attracting more citations. However, no statistically significant difference in AAS was identified. While the *Ann Rheum Dis* guidelines have greater impact, the *Arthritis Rheumatol* guidelines have similar visibility on online and/or SoMe platforms.

### Influence of publication period

A temporal comparison demonstrated that older guidelines (pre-2015) had significantly higher citation counts than those published in the last decade, which is possibly due to longer citation accumulation time. In contrast, guidelines published after 2015 had significantly higher median AAS values, possibly due to improved online engagement and public awareness in recent times [18, 19].

### Top-performing guidelines

Several guidelines stood out for their academic and digital influence. The 2015 ACR guideline for rheumatoid arthritis received considerable attention, evidenced by 2590 citations, underscoring its significant impact within the academic community. The highest AAS values were recorded for guidelines on rheumatoid arthritis and osteoarthritis, reflecting their substantial clinical significance and public engagement [12]. Additionally, three guidelines ranked among the top five for both citation and AAS values, illustrating that certain clinical practice guidelines achieved dual success in academic influence and digital visibility. This suggests that academic recognition and public dissemination on digital platforms can efficiently coexist in impactful rheumatology guidelines.

### Correlation trends

The correlation analysis demonstrated complex connections between traditional citation metrics and altmetric indices. Statistically significant positive correlations were found between Scopus citation counts and AAS, news outlets mentions, and Facebook mentions, suggesting that items receiving more online attention are more likely to be referenced in the literature. Nonetheless, this correlation was not strong, substantiating the concept that altmetrics and traditional citations represent distinct dimensions of impact [20].

Stronger correlations were found between Scopus citations and both Mendeley bookmarkings and Dimensions citation counts, suggesting that the Mendeley metric can be a reliable early predictor of academic interest and prospective citations. This is consistent with prior research suggesting that Mendeley readership indicated sustained scholarly impact, possibly due to its predominant utilization by academics, researchers, and health professionals [21].

The Mendeley bookmarking count showed statistically significant positive correlations with various altimetric parameters, including AAS, news outlets, Facebook, and X (Twitter) user activities. These data indicate that Mendeley bookmarking is bridging data, representing both initial scholarly interest and further digital distribution. Widespread use of Mendeley by academics and health professionals to gather materials may suggest hidden citation potential and general professional interest, reflected in higher bookmarking/readership numbers [22].

No significant correlation was recorded between Scopus citations and X (Twitter) activity, suggesting that visibility on social microblogging platforms does not inherently precipitate mainstream academic adoption. This phenomenon may reflect transient and informal character of X (Twitter) activities, wherein mentions might imply immediate acknowledgment or discussion, yet they often lack enduring academic engagement. These data underscore that altmetrics and traditional citations represent distinct aspects of influence/impact, emphasizing the necessity of interpreting them in a complementary, rather than interchangeable, manner.

## Limitations

Admittedly, there are some limitations. The analysis focused only on two global rheumatology journals, which led to the exclusion of relevant guidelines from other sources. Secondly, AAS is a dynamic metric, subject to temporal variations. Although all data were obtained on a single day, the outcomes reflect a snapshot rather than a longitudinal trend. Relying exclusively on Scopus as the citation source may constrain the scope, as alternative databases might yield different citation counts. Furthermore, specific clinical practice guidelines, particularly those collaboratively created by EULAR and ACR, are published simultaneously in *Ann Rheum Dis* and *Arthritis Rheumatol*. We did not specifically consider this dual-publication approach in our analysis.

### Improving dissemination strategies for rheumatology guidelines

As SoMe increasingly influences medical communication, attention has transitioned to the editorial strategies that govern content distribution. SoMe editors affiliated with journals are essential in augmenting the visibility and impact of guidelines. In addition to the appointment of SoMe editors, establishing active accounts on platforms such as Instagram, widely used by rheumatologists, can further enhance dissemination efforts. The visual tools provided by this platform, including infographics and brief videos, effectively engage professionals and the general public [23–25]. This matter is especially pertinent in light of our findings, illustrating the significance of integrated strategies across traditional and SoMe. Specifically, certain guidelines demonstrated both substantial academic impact and considerable online visibility.

## Conclusion

This study underscores the changing dynamics of impact in rheumatology clinical practice guidelines. While older guidelines continue to top traditional citation rankings, recent documents are increasingly disseminated and discussed on online and social networking platforms, adding to altmetric values. The existence of guidelines that attain elevated standings in citation and altmetric indices signifies the advent of a dual-impact paradigm wherein scientific credibility and digital dissemination intricately coexisted. The correlations between Mendeley bookmarking data and both academic and altmetric measures underscore the significance of early awareness among professional communities as a possible catalyst for sustained effect. Journal editors and publishers should adopt a cohesive strategy that utilizes academic networks and online interaction platforms to optimize future rheumatology practice guidelines’ clinical and societal influence. Enforcing such a strategy may translate into publishing more influential and broadly used evidence-based documents.

## Data Availability

Raw data can be provided upon reasonable request from the corresponding author.
